# Body mass index in early-pregnancy and selected maternal health outcomes: Findings from two cohorts in Bangladesh

**DOI:** 10.7189/jogh.10.020419

**Published:** 2020-12

**Authors:** Monjur Rahman, Syed Moshfiqur Rahman, Jesmin Pervin, Shaki Aktar, Shams El Arifeen, Anisur Rahman

**Affiliations:** 1 Maternal and Child Health Division, International Centre for Diarrhoeal Disease Research, Bangladesh; 2 International Maternal and Child Health, Department of Women’s and Children’s Health, Uppsala University, Uppsala, Sweden

## Abstract

**Background:**

Maternal nutrition is one of the most influential factors that affect the health of the mother and her offspring and remains a significant public health challenge globally. There is a lack of studies evaluating the trends of maternal nutrition and its impact on the burden of pregnancy complications from low-income countries, including Bangladesh. We aimed to determine the burden of early-pregnancy nutrition status based on body mass index (BMI), and the associations of nutritional status with pregnancy-induced hypertension (PIH), cesarean section (CS) delivery, perineal tear and postpartum hemorrhage (PPH) in a rural area in Bangladesh.

**Methods:**

This prospective study analyzed data from two cohorts: the Maternal, Neonatal, and Child Health (MNCH) project carried out from January 2008 to June 2010, and the Preterm and Stillbirth Study, Matlab (PreSSMat) conducted from October 2015 to March 2018. In total, information of 9287 women who gave birth from the two cohorts was available for analysis. Early-pregnancy BMI was categorized into underweight, normal-weight, and overweight groups. The change in the burden of malnutrition between two cohort periods and the associations between women’s BMI and maternal health outcomes were presented in odds ratios (ORs) with their 95% confidence interval (CI).

**Results:**

Between the two cohort periods, the prevalence of underweight decreased from 17.5% to 15.4%, and overweight increased from 10.8% to 20.9%. The risk of being overweight in pregnant women was about two times (OR = 2.19; 95% CI = 1.94-2.46) higher in the PreSSMat cohort than in the MNCH cohort. After multivariate-adjustment for socio-demographic factors, the pooled ORs of PIH, CS delivery, perineal tear, and PPH were 2.41 (95% CI = 1.95-2.99), 2.12 (95% CI = 1.86-2.41), 2.46 (95% CI = 1.54-3.92), and 1.68 (95% CI = 1.12-2.53), respectively, in women with overweight compared to the normal-weight group.

**Conclusions:**

The results confirmed the existence of a double burden of malnutrition in rural women in Bangladesh. Women with overweight had an increased risk of selected pregnancy complications. The findings call for the adoption of appropriate prenatal counseling and preparedness tailored to women’s nutritional status to prevent possible adverse health outcomes.

Maternal nutrition is one of the most influential factors that affect the health of mothers and their offspring [1,2]. In population-based studies, nutrition status is commonly assessed by body mass index (BMI) that is calculated by weight in kilograms divided by the square of the height in meters (kg/m^2^). Due to immediate-, short-, and long-term health effects on mothers and their children, both underweight (BMI<18.5) and overweight (BMI≥25.0) are recognized as important public health issues in all countries across the world [3,4]. The achievement of the Sustainable Development Goals (SDGs) 2 and 3, ending all forms of malnutrition and ensuring healthy lives and well-being for all ages, respectively, rely on how a country adopts strategies to prevent malnutrition and its associated health impacts [5].

It is estimated that about 153.8 million women of reproductive age have been suffering from underweight worldwide [6]. Almost half of these women live in South Asia, particularly in Bangladesh, India, and Pakistan [7,8]. Globally, the prevalence of maternal underweight has declined from 11.6% in 2000 to 9.7% in 2016 [6]. In Bangladesh, the proportion of women with underweight decreased from 52% in 1996 to 19% in 2014 [9]. Although the data indicate a substantial decline in prevalence, the current burden is still high compared to many low- and middle-income countries.

At the other end of the malnutrition spectrum, overweight has been an alarming health concern in high-income countries for decades [10,11]. Due to rapid economic development, many low-income countries are now in a phase of nutrition and epidemiologic transitions that have led to an increase in overweight or obesity in women of reproductive age at a population level [12]. The proportion of overweight has increased from 29% in 1980 to 38% in 2013, globally [13]. In Bangladesh, the burden of overweight also increased from 3% in 1996 to 24% in 2014 [9].

The associations of high BMI with adverse maternal health outcomes in pregnancies are well documented from high-income settings [14]. Overweight in pre- or early-pregnancy is associated with high risks of pregnancy-induced hypertension (PIH), and cesarean section (CS) delivery [15-19]. Studies also have investigated the associations between BMI and other maternal morbidities, including perineal tear [20-23] and postpartum hemorrhage (PPH) [17,24,25]. However, the reported associations are inconsistent. A few studies have reported that overweight is protective against perineal trauma [20,22], while others have suggested high risk for perineal injury [21,23]. Several studies, mainly from high-income countries, have investigated the impact of obesity on PPH and have reported a small [24,26] to a moderate increase of PPH odds [27,28] in obese women compared to the normal-weight women. However, few study findings did not support the associations [29,30].

Studies on trends in maternal malnutrition and its association with health outcomes during pregnancy are scarce from low-income countries. The available studies are cross-sectional [17], and based on data from the National Demographic Health Surveys, or hospital-based information with small sample size [31-33]. There are also inconsistencies in reported associations between maternal BMI and the above pregnancy complications, thus warranting more evaluations. Therefore, our primary objective was to evaluate the associations between early-pregnancy BMI and selected maternal health outcomes based on two well-characterized cohorts conducted in rural Matlab, Bangladesh. Further, we also assessed the burden of malnutrition in women in the early gestational period over time.

## METHODS

### Study settings, design and study subjects

The study site is located in Matlab Upazila in Chandpur district, Bangladesh. International Centre for Diarrhoeal Disease Research, Bangladesh (icddr,b) has been running a Health and Demographic Surveillance System (HDSS) in a population of about 220 000 since 1966. This study was conducted in half of the HDSS area, where icddr,b provides health services to women of reproductive age and the children below five years. The study area has four administrative blocks, and each block has a health sub-center where midwives provide 24-hour maternal and child health services. A hospital run by icddr,b, and located in Matlab Township, provides support as a referral facility for women and children from the community and sub-centers. However, the Matlab Hospital provides only basic obstetric care by nurses and medical officers. Due to the lack of cesarean facility, a woman who requires CS needs a referral to a public health facility or private clinic with comprehensive obstetric care provision.

In the present paper, we used available information from two cohorts – the Maternal, Neonatal, and Child Health (MNCH) project, and the Preterm and Stillbirth Study, Matlab (PreSSMat). In addition to this, the routine data collected by the HDSS was also used. The MNCH project aimed to strengthen the existing maternal and child health services along the continuum of pregnancy, delivery, and postpartum periods. The study was carried out between January 2008 and June 2010 [34]. The purpose of the PreSSMat project was to establish a cohort to collect related information to understand the biological determinants of preterm births [35]. The PreSSMat was conducted between October 2015 and March 2018.

In the current study, the women were considered eligible for analyses if the following criteria were met: (i) the women were identified as pregnant by the HDSS, (ii) the pregnant women provided consents for participation in the MNCH or PreSSMat study, and (iii) the anthropometric measurements were available before 20 gestation weeks (GWs) of age.

### Data collection

The exposure and outcome information was obtained from the MNCH and PreSSMat databases, while the covariate information was extracted from the HDSS databases. The HDSS recorded socio-demographic characteristics and updated the information through household visits of community health workers (CHWs) every two-month. During household visits in the study area, CHWs asked women of reproductive age if they had missed their periods. Women who reported menstrual period being at least two weeks overdue were invited to take a urine pregnancy test. Women with positive pregnancy test results were requested to attend the respective sub-center or Matlab Hospital for further assessment by ultrasound. A woman, who provided her consent for participation in the study, was invited to visit to undergo four antenatal care services and use icddr,b facilities for delivery, and postpartum care. Medical history, physical examination, and clinical data collection were similar for both cohorts and were collected during each contact.

#### Exposure

The exposure variable was the women’s BMI, which was calculated from the mother's weight and height collected before 20 GWs of age. Maternal weight was measured by using a bathroom scale (SECA, Uniscale, Hamburg, Germany) with a precision of 100g. Height was measured with a locally made wooden scale (precision 0.1cm). The BMI was categorized as underweight (BMI<18.5 kg/m^2^), normal-weight (BMI = 18.5-24 kg/m^2^), and overweight (BMI≥25 kg/m^2^). Overweight group is further divided into pre-obese (BMI = 25-29 kg/m^2)^ and obese (BMI≥30 kg/m^2^) [36].

#### Outcomes

We evaluated the selected maternal health outcomes during pregnancy, such as PIH, CS, perineal tear, and PPH. In the MNCH and PreSSMat cohorts, PIH was classified as chronic, gestational, preeclampsia/eclampsia, or super-imposed preeclampsia with chronic hypertension. Chronic hypertension was defined when the woman had high blood pressure (systolic blood pressure ≥140 mm Hg or diastolic blood pressure ≥90 mm Hg) before pregnancy or early in pregnancy (before 20 GWs) and continued to have it after delivery. If a previously normotensive woman developed high blood pressure with no protein in urine after 20 GWs, it was considered to be gestational hypertension. Preeclampsia was defined as gestational hypertension with proteinuria 2+ or higher on dipstick testing. Eclampsia was considered as the onset of convulsion or seizures in a woman with preeclampsia. Super-imposed preeclampsia with chronic hypertension was identified if the diagnosed women had been with chronic hypertension already before the pregnancy and then developed preeclampsia during pregnancy. The mode of delivery was classified as vaginal or CS delivery. A perineal tear was defined as laceration of the skin, which extends beyond fourchette, perineal skin, and vaginal mucosa – to perineal muscles and fascia, or tear of the external of the anal sphincter. A first-degree perineal tear was defined as an injury to the vaginal mucosa and connective tissue. A second-degree perineal tear was an injury to the perineum involving vaginal mucosa and perineal muscles without involving the anal sphincter. When the injury involved the anal sphincter complex, it was considered to be a third-degree tear. PPH was defined as the loss of more than 500 ml of blood within the first 24 hours following childbirth. The attending midwife or medical officer diagnosed PPH by estimating the number of pads soaked by blood in 24 hours of delivery or observing if a pad or the cloths soaked within 5 minutes or by the constant trickling of blood [37].

#### Covariates

The mother’s age was calculated by subtracting the date of birth from the date of the last menstrual period (LMP) and was expressed in years. Parity was defined as the number of live birth or stillbirth born before the current pregnancy. Maternal education was measured by the number of completed years in a formal school. We assessed the wealth status by generating scores through principal components analysis. It was based on the ownership of household assets, such as consumer items (eg, television, almirah, mobile phone, and other valuable items), dwelling characteristics (wall and roof materials), type of drinking water, and toilet facilities. The generated scores were then divided into quintiles, where one represented the poorest and five, the wealthiest.

### Statistical analysis

Descriptive statistics such as frequency, mean (Standard deviation, SD), median, and proportions were used to characterize the study participants. In the analyses, maternal age was categorized into <20, 20-24, 25-29, or ≥30 years, parity into 0, 1-2, or ≥3, mother’s education into no education (0 years), primary (1-5 years), or secondary or higher (≥6 years). Associations of available covariates, exposure and outcomes between two cohorts were assessed by χ^2^ tests. We graphically presented the trend of nutritional status by BMI and the outcomes. The changes in proportions of women with underweight and overweight compared to the women with normal-weight between MNCH and PreSSMat cohorts were assessed by multinomial logistic regression analysis. The associations between early-pregnancy BMI and maternal health outcomes were assessed by using binary logistic regression separately for each cohort. Covariates associated with exposure and outcome at *P* < 0.2 were included in the logistic regression analysis, while a backward stepwise approach was used to identify those independently associated with the outcome of interest (*P* < 0.05) to keep in the final multivariate model. Furthermore, the effect estimates from the multivariate-adjusted regression models in each cohort were pooled to obtain the summary risk estimates with the use of an inverse variance-weighted meta-analysis. We used both random- and fixed-effect models. However, we presented the result from the fixed-effect model due to the observed *P* value of heterogeneity at >0.1. The results of regression analyses were presented by crude and adjusted odds ratios (ORs) with their 95% confidence intervals (CI). We used Stata version 13 for all statistical analyses (StataCorp, College Station, TX, USA).

### Ethical consideration

Ethical approval was obtained from the Research and Ethical Review Committees of the International Centre for Diarrhoeal Disease Research, Bangladesh (icddr,b) for both of the cohort studies. All the participants provided informed written consent before study enrollment, and each individual was assigned an anonymous participant identification code.

## RESULTS

**Figure 1:**
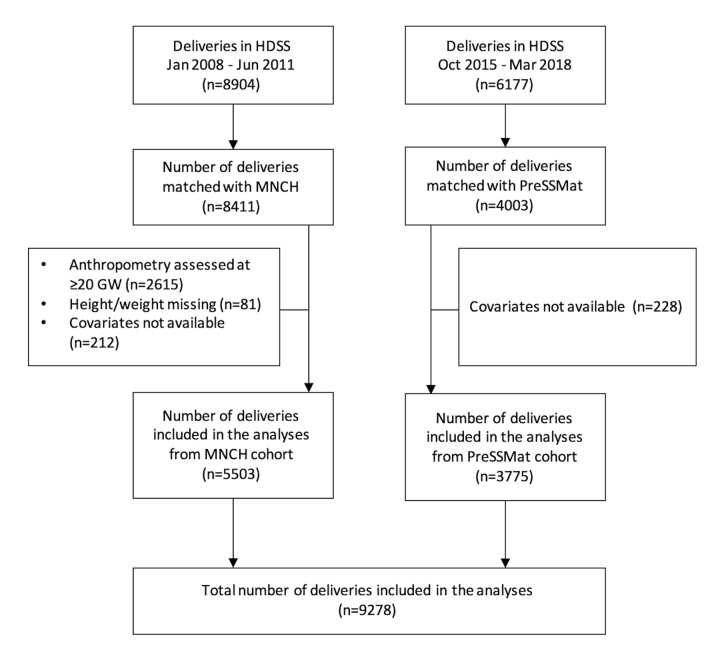
Study profile. HDSS, health and demographic surveillance system; MNCH, maternal, neonatal, and child health, PreSSMat, preterm and stillbirth study, Matlab.

Out of 15 081 deliveries recorded in the HDSS databases, 12 414 (82.3%) deliveries were available in the MNCH and PreSSMat databases, where women provided consents for participation. After excluding the pregnant women who appeared in the facilities at ≥20 GWs for the first antenatal care service, and the women with missing covariate information, 9278 (5503 from MNCH and 3775 from PreSSMat) deliveries were available and thus included in the analysis (**Figure 1**). The mean (SD) gestational ages of weight measurements were 16.6 (1.1) and 12.9 (1.9) weeks for the MNCH and PreSSMat cohorts, respectively.

The background characteristics of the study women from both cohorts are presented in Table 1. The mean ages of women in the MNCH and PreSSMat cohorts were 25.8 (SD = 5.9), and 25.4 (SD = 5.9) years. The mean and median years of school attendance were 6.2 (SD = 3.6) and seven years, respectively, for the MNCH cohort. For the PreSSMat cohort, the figures were 7.5 (SD = 3.7) and eight years, respectively. Overall, compared to the MNCH cohort women, the women from the PreSSMat cohort had higher age, lower parity, and higher education attainment by years of school attendance (**Table 1**).

**Table 1:** Background characteristics of study participants in the two cohorts in Matlab, Bangladesh

	All participants	MNCH	PreSSMat	
	**N = 9278**	**n = 5503**	**n = 3775**	*P*-value*
**Maternal age (year), n (%):**				
<20	1730 (18.6)	1035 (18.8)	695 (18.4)	0.018
20-24	2997 (32.3)	1837 (33.4)	1160 (30.7)	
25-29	2361 (25.4)	1378 (25.0)	983 (26.0)	
≥30	2190 (23.6)	1253 (22.8)	937 (24.8)	
**Parity, n (%):**				
0	3311 (35.7)	1924 (35.0)	1387 (36.7)	<0.001
1	5197 (56.0)	3053 (55.5)	2144 (56.8)	
≥2	770 (8.3)	526 (9.6)	244 (6.5)	
**Education (year), n (%):**				
0	1124 (12.1)	717 (13.0)	407 (10.8)	<0.001
1-5	2186 (23.6)	1528 (27.8)	658 (17.4)	
≥6	5968 (64.3)	3258 (59.2)	2710 (71.8)	
**Wealth quintile, n (%):**				
1-Poorest	1429 (15.4)	822 (14.9)	607 (16.1)	0.470
2	1633 (17.6)	961 (17.5)	672 (17.8)	
3	1829 (19.7)	1079 (19.6)	750 (19.9)	
4	2064 (22.2)	1243 (22.6)	821 (21.7)	
5-Wealthiest	2323 (25.0)	1398 (25.4)	925 (24.5)	

MNCH – Maternal, Neonatal, and Child Health, PreSSMat – Preterm and Stillbirth Study, Matlab

*Significance levels by χ^2^ tests for background characteristics between MNCH and PreSSMat cohort.

**Figure 2:**
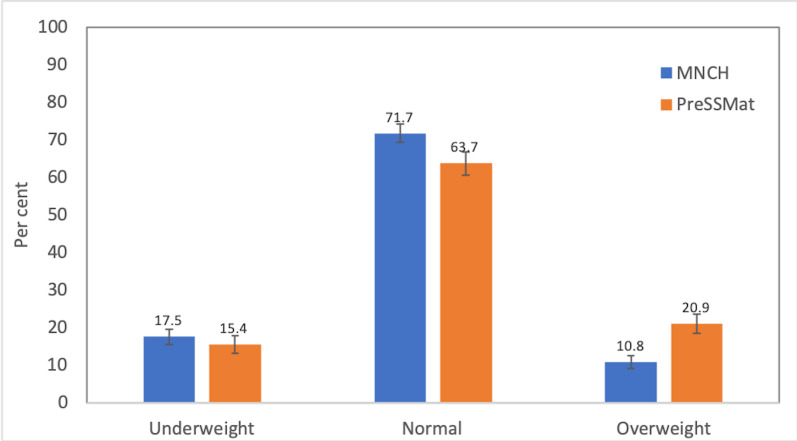
Change in prevalence of underweight and overweight pregnant women. MNCH, maternal, neonatal, and child health; PreSSMat, preterm and stillbirth study, Matlab. Error bars represent 95% confidence interval bands.

The mean weight and height of the women in MNCH cohort were 47.8 (SD = 7.6) kg, and 150.5 (SD = 5.7) cm, respectively, whereas these were 50.9 (SD = 9.0) and 151.8 (SD = 5.2) in the PreSSMat cohort, respectively. The mean BMI was 21.1 (SD = 3.1) and 22.1 (SD = 3.6) kg/m^2^ in MNCH and PreSSMat cohorts. Due to a small number of women with obesity (53 in MNCH, 109 in PreSSMat), we merged the pre-obese and obese groups as an overweight group. Maternal nutritional status, assessed by BMI, indicated that the proportion of underweight women decreased marginally – from 17.5% in the MNCH cohort to 15.4% in the PreSSMat cohort. In contrast, the proportion of overweight increased remarkably from 10.8% to 20.9% between the two study periods (**Figure 2**).

**Figure 3:**
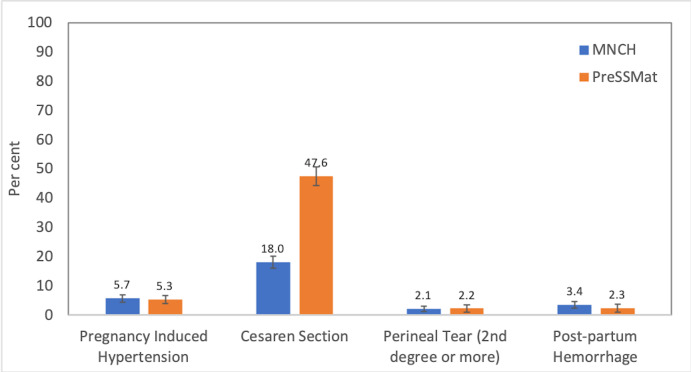
Proportion of maternal outcomes by cohort. MNCH, maternal, neonatal and child health; PreSSMat, preterm and stillbirth study, Matlab. Error bars represent 95% confidence interval bands.

**Figure 3** presents the proportion of maternal health outcomes in the two cohorts. The CS rate was very high in this population. The rate significantly increased from about 18% in the MNCH cohort to about 48% in the PreSSMat cohort (**Figure 3**).

The associations between nutritional status during early-pregnancy and the available covariates are presented by each cohort (Table S1 in the **Online Supplementary Document**). In both cohorts, all the covariates were found to be significantly associated with nutritional status during early pregnancy (Table S1 in the **Online Supplementary Document**).

We assessed the associations of maternal health outcomes with available covariates in MNCH and PreSSMat cohorts (Table S2 and S3 in the **Online Supplementary Document**). The associations with those socio-demographic factors differ between cohorts. Based on the criteria to determine the potential confounders in the analysis plan, we assessed the covariates’ associations with exposures and outcomes for inclusion in the multivariate logistic regression model (Table S4 **Online Supplementary Document**).

In the multinomial logistic regression, we did not observe any change in underweight (OR = 0.99, 95% CI = 0.88-1.11) between the two cohorts. However, the risk of the women being overweight was about two times higher (OR = 2.19, 95% CI = 1.94-2.46) in the PreSSMat cohort compared to those in the MNCH cohort.

Maternal overweight was found associated with increased risks of PIH, CS delivery, and perineal tear in both cohorts (**Table 2** and **Table 3**). However, for PPH, the risk was only observed in the MNCH cohort (**Table 2**). The women in the underweight group were less likely to have CS in the MNCH cohort, and no association with other maternal outcomes was observed (**Table 2**). In the PreSSMat cohort, decreased odds of PIH and CS deliveries were observed in women with underweight (**Table 3**).

**Table 2:** Associations between early-pregnancy body mass index and maternal health outcomes in the MNCH cohort in Matlab, Bangladesh

	Pregnancy-induced hypertension		Cesarean section		Perineal tear (2^nd^ degree or more)*		Postpartum hemorrhage*
**BMI category†**	**OR (95% CI)**	**aOR (95% CI) ‡**	**OR (95% CI)**	**aOR (95% CI)**§	**OR (95% CI)**	**aOR (95% CI)‖**	**OR (95% CI)**¶
Underweight	0.78 (0.55-1.11)	0.78 (0.55-1.11)	0.58 (0.47-0.72)	0.56 (0.45-0.70)	1.11 (0.65-1.88)	1.01 (0.60-1.73)	0.90 (0.58-1.39)
Normal-weight**	1	1	1	1	1	1	1
Overweight	2.52 (1.90-3.37)	2.59 (1.94-3.45)	2.53 (2.10-3.05)	2.73 (2.23-3.32)	2.08 (1.17-3.69)	2.84 (1.57-5.12)	1.97 (1.25-3.10)

BMI – body mass index, OR – odds ratio, aOR – adjusted odds ratio; CI – confidence interval

*Analysis limited to women with vaginal delivery

†BMI category: underweight (<18.5 kg/m^2^), normal-weight (18.5-24 kg/m^2^), and overweight (≥25 kg/m^2^)

‡Adjusted for maternal education

§Adjusted for maternal parity, education and wealth quintile

‖Adjusted for maternal parity

¶No covariate associated with exposure and outcome, thus crude OR is presented

**Reference category.

**Table 3:** Associations between early-pregnancy body mass index and maternal health outcomes in the PreSSMat cohort in Matlab, Bangladesh

	Pregnancy-induced hypertension		Cesarean section		Perineal tear (2^nd^ degree or more)*		Postpartum hemorrhage*
BMI category†	**OR (95% CI)**	**aOR (95% CI)‡**	**OR (95% CI)**	aOR* (95% CI)§	**OR (95% CI)**	**aOR (95% CI)‖**	**OR (95% CI)**¶
Underweight	0.53 (0.31-0.92)	0.47 (0.27-0.81)	0.74 (0.61-0.89)	0.69 (0.57-0.84)	1.12 (0.50-2.50)	0.97 (0.43-2.20)	1.85 (0.94-3.64)
Normal-weight**	1	1	1	1	1	1	1
Overweight	1.95 (1.43-2.66)	2.21 (1.59-3.05)	1.69 (1.43-1.99)	1.76 (1.48-2.09)	1.56 (0.74-3.27)	1.94 (0.91-4.15)	0.92 (0.38-2.26)

BMI – body mass index, OR – odds ratio, aOR – adjusted odds ratio, CI – confidence interval

*Analysis limited to women with vaginal delivery.

†BMI category: underweight (<18.5 kg/m^2^), normal-weight (18.5-24 kg/m^2^), and overweight (≥25 kg/m^2^).

‡Adjusted for maternal parity and wealth quintile.

§Adjusted for maternal parity, age and wealth quintile.

‖Adjusted for maternal age.

¶No covariate associated with exposure and outcome, thus crude OR is presented.

**Reference category.

The meta-analyses demonstrated that compared to the normal-weight group, the pooled adjusted ORs in the overweight women group for PIH, CS, perineal tear and PPH were 2.41 (95% CI = 1.95-2.99), 2.12 (95% CI = 1.86-2.41), 2.46 (95% CI = 1.54-3.92), and 1.68 (95% CI = 1.12-2.53), respectively (**Table 4**). The women in the underweight group were less likely to have PIH (OR = 0.67, 95% CI = 0.50-0.90) and CS deliveries (OR = 0.63, 95% CI = 0.55-0.73) only (**Table 4**).

**Table 4:** Associations between early-pregnancy body mass index category and maternal health outcomes by meta-analyses based on the effect estimated in multivariable-adjusted regression in the MNCH and PreSSMat cohorts in Matlab, Bangladesh

BMI category*	Pregnancy-induced hypertension	Cesarean section	Perineal tear (2^nd^ degree or more)†	Postpartum hemorrhage†
	OR (95% CI)‡	OR (95% CI)‡	OR (95% CI)‡	OR (95% CI)‡
Underweight	0.67 (0.50-0.90)	0.63 (0.55-0.73)	1.00 (0.64-1.56)	1.11 (0.77-1.61)
Normal-weight§	1	1	1	1
Overweight	2.41 (1.95-2.99)	2.12 (1.86-2.41)	2.46 (1.54-3.92)	1.68 (1.12-2.53)

BMI – body mass index, OR – odds ratio, CI – confidence interval

*BMI category: underweight (<18.5 kg/m^2^), normal-weight (18.5-24 kg/m^2^), and overweight (≥25 kg/m^2^).

†Analysis limited to women with vaginal delivery.

‡Pooled adjusted odds ratio using fixed effect model.

§Reference category.

## DISCUSSION

Based on the data from two prospective cohorts, the present study has found the co-existence of underweight and overweight pregnant women, and the associations of high early-pregnancy BMI with increased risk of adverse maternal health outcomes in a rural area in Bangladesh. We observed no changes in the prevalence of underweight. However, the burden of overweight increased about two times in the later cohort than the earlier one. Compared to the women in the normal-weight group, we found the overweight women had increased risk of PIH, CS delivery, perineal tear, and PPH.

The double burden of malnutrition observed in our study is consistent with studies conducted in other South Asian countries, including India and Pakistan [38]. Several studies have reported that both underweight and overweight are prevalent in the Bangladeshi population. A recent cross-sectional survey of women of reproductive ages reported the prevalence of underweight and overweight to be 32% and 14% in 2007; and 21% and 26% in 2014, respectively [31]. Although the burden of overweight was similar, the proportion of underweight was lower in our study sample. This variation may have arisen due to anthropometric measurements after delivery in the survey study. The Bangladesh Demographic and Health Survey (BDHS) has reported that the proportion of underweight among the pregnant population is 18%, consistent with our finding [9].

There is a scarcity of studies evaluating the association of maternal pre- or early-pregnancy overweight with pregnancy complications in low-income countries, including Bangladesh [17,32,38]. Thus far, only one study has used information from the BDHS, in this case, from the years 2011 and 2014 to report associations between maternal BMI and increased risks of hypertension and CS [32]. However, that study had several limitations, the BMI of women were assessed at the survey interview, and the outcome information was based on the data collection by five years recall [32]. Overweight has consistently been reported to be associated with a high risk of PIH and CS deliveries. The estimated risk increases observed at two to three times higher than the women with normal-weight in those studies [17,39,40] are comparable with the risks observed in our study.

The reported relationships between overweight and perineal tear and postpartum hemorrhage are inconsistent. Only a few studies are available that evaluated the impact of obesity on perineal trauma. However, in contrast to our findings, the studies reported obesity as a protective factor against both mild and severe forms of perineal injury [20,22]. The risk estimate of perineal tear in the study is consistent with the studies conducted in Slovenia and Saudi Arabia [21,41]. A recent hospital-based cohort study conducted in California, US, reported a small but significant increase of PPH (OR = 1.06, 95% CI = 1.04-1.08) [24]. Another study that used the birth registry data in Sweden also reported a similar risk increase for PPH (OR = 1.08, 95% CI = 1.04-1.11) in overweight women who delivered vaginally [26]. The observed OR in our study was similar to the studies conducted in New Zealand and the UK, which reported the estimated ORs for PPH to be 2.11 (95% CI = 1.84-2.89) and 1.4 (99% CI: 1.3-1.6), respectively [27,28].

The increased maternal overweight trends in our study may be explained by consistent economic growth over the last decade in Bangladesh. It is estimated that the country’s per-capita GDP increased from US$ 780 in 2009-10 to US$ 1544 in the 2016-2017 fiscal year [42]. There are also changes in food habits, social, and lifestyle patterns, leading to more physical inactivity in rural and urban areas. Even with the rapid social changes and economic development, the distribution of wealth is unequal within the population. This inequality explains the sizable proportion of undernourished women in this rural community and other parts of the country [31,43].

The mechanisms behind the association of malnutrition with morbidities in pregnancies are not fully elucidated. Hypertensive disorders due to obesity are possibly mediated through activation of the sympathetic nervous system, and renal impairment, and also by inflammatory mediators [44-46]. Other possible mechanisms linked with hypertension in pregnancy are oxidative stress, insulin resistance, and endothelial dysfunctions [47]. Lifestyle changes such as poor food-habits, smoking, and other stressful conditions were reported as possible mediators of the association between overweight and PIH in women [48]. High CS rates in overweight or obese women may be related to slow progress of labor, increased labor induction, and associated co-morbid conditions such as diabetes mellitus, hypertension, and macrosomia [49]. The protective effect of obesity against severe perineal morbidity is speculated since increased adipose tissue in the pelvic floor increases the perineum ability to stretch and also widens the gap between the vaginal canal and rectum [20,22]. Perhaps, the women from high BMI group in our study delivered in a lithotomy position, and did not follow the appropriate pushing techniques during the second stage of labor, resulting in increased frequency of perineal injury. Previous studies have attributed the increased risk of PPH to less contractility of the uterine muscle [27,50,51] and increased intervention during labor in obese women [52].

The present study has several strengths. The analyses were based on two prospectively collected cohorts of pregnant women, a reasonable number of sample sizes for each cohort, and a high number of contacts during antenatal and delivery periods for obtaining the outcome and related information. Further, the relevant socio-demographic variables were available for making adjustments to address confounding issues.

However, several limitations should be considered when interpreting the study findings. The BMI was assessed based on weight measurement at mean gestational weeks of 16.6 (1.1) and 12.9 (1.9), respectively, in the MNCH and PreSSMat cohorts. Due to limited weight gain before 20 weeks of gestation, this variation is unlikely to affect the measurement of malnutrition. A substantial proportion of women were excluded (25.3%) from the analyses who participated in two cohorts. However, we did not find any significant difference in outcomes except CS (Table S5 in the **Online Supplementary Document**). This non-significant distribution of outcomes and also a very high participation rate (82%) in the cohorts support the validity and representativeness of the study results to the population level, respectively. Like previous studies, we were not able to measure the amount of blood loss during the postpartum period. In both cohorts, we observed similar pattern of risk increase for PIH, CS, and perineal tear, but not for PPH. The subjective measurement of blood loss may compromise the internal validity of PPH’s assessment in our study.

In this study, we investigated selected maternal health outcomes, which have a significant contribution to the health and survival of mothers and their offspring. PIH and PPH are reported as the major causes of maternal mortality in developing countries, including Bangladesh [53]. The burden of CS is also a public health challenge, as demonstrated by a recent increase in CS rate at the population level in South Asian countries. The BDHS reported that the national CS rate increased more than 5-fold in a decade, from 4% in 2004 to 23% in 2014 [9]. It is also documented that CS may increase complications during subsequent pregnancies [54]. Perineal trauma is one of the most common morbidities in birthing women and is associated with short- and long-term health consequences such as increased hemorrhage, puerperal infection, urinary and fecal incontinence, and dyspareunia [55]. Further research should focus on understanding the causal link between high BMI and these pregnancy complications, and more studies are needed from low-income countries.

In conclusion, we have reported a double-burden of malnutrition in a rural area in Bangladesh. While the burden of underweight women has remained static, the proportion of overweight women has increased sharply in a short period, even in a rural community. Further, overweight women had increased proportions of PIH, CS delivery, perineal tear, and PPH. All these health conditions have significant short- and long-term health impacts on women and their offspring. The study results warrant appropriate counseling and preparedness tailored to women’s nutritional status to prevent possible adverse health outcomes in women and children.

## Additional material

Online Supplementary Document
