# Supplementary material for: Body mass index in early-pregnancy and selected maternal health outcomes: Findings from two cohorts in Bangladesh
Source: J Glob Health. 2020 Sep 23;10(2):020419. doi: 10.7189/jogh.10.020419 (PMC7568936; doi:10.7189/jogh.10.020419)
Supplement: Online Supplementary Document [file jogh-10-020419-s001.pdf]

Supplementary tables

Table S1. Associations between background characteristics and body mass index category in two cohorts in Matlab, Bangladesh.

	MNCH				PreSSMat			
	**Underweight**	**Normal-weight**	**Overweight**		**Underweight**	**Normal-weight**	**Overweight**	
	**n(%)**	n(%)	n(%)	*P*-value*	n(%)	n(%)	n(%)	*P*-value*
Maternal age								
<20	219 (22.8)	785 (19.9)	31 (5.2)	<0.001	201 (34.6)	443 (18.4)	51 (6.5)	<0.001
20-24	381 (39.6)	1317 (33.4)	139 (23.4)		212 (36.5)	763 (31.7)	185 (23.4)	
25-29	207 (21.5)	980 (24.8)	191 (32.2)		98 (16.9)	644 (26.8)	241 (30.5)	
≥30	155 (16.1)	866 (21.9)	232 (39.1)		70 (12.0)	554 (23.0)	313 (39.6)	
**Parity**								
0	397 (41.3)	1417 (35.9)	110 (18.5)	<0.001	342 (58.9)	900 (37.4)	145 (18.4)	<0.001
1-2	498 (51.8)	2161 (54.7)	394 (66.4)		220 (37.9)	1371 (57.0)	553 (70.0)	
≥3	67 (7.0)	370 (9.4)	89 (15.0)		19 (3.3)	133 (5.5)	92 (11.6)	
Education (year)								
0	136 (14.1)	515 (13.0)	66 (11.1)	<0.01	75 (12.9)	266 (11.1)	66 (8.4)	<0.05
1-5	273 (28.4)	1125 (28.5)	130 (21.9)		99 (17.0)	436 (18.1)	123 (15.6)	
>10	553 (57.5)	2308 (58.5)	397 (66.9)		407 (70.1)	1702 (70.8)	601 (76.1)	
Wealth quintile								
1- Poorest	180 (18.7)	584 (14.8)	58 (9.8)	<0.001	109 (18.8)	407 (16.9)	91 (11.5)	<0.001
2	170 (17.7)	711 (18.0)	80 (13.5)		125 (21.5)	450 (18.7)	97 (12.3)	
3	205 (21.3)	775 (19.6)	99 (16.7)		144 (24.8)	476 (19.8)	130 (16.5)	
4	199 (20.7)	904 (22.9)	140 (23.6)		94 (16.2)	535 (22.3)	192 (24.3)	
5-Wealthiest	208 (21.6)	974 (24.7)	216 (36.4)		109 (18.8)	536 (22.3)	280 (35.4)	

MNCH, Maternal, Neonatal, and Child Health; PreSSMat, Preterm and Stillbirth Study, Matlab
Body mass index category: underweight (<18.5 kg/m^2), normal-weight (18.5-24 kg/m^2), and overweight (≥25 kg/m^2)
*level of significance by χ^2 tests

Table S2. Association between background factors and pregnancy complications of MNCH cohort in Matlab, Bangladesh

	**PIH**			**CS**			**Perineal tear (2nd degree or more)***			**PPH***		
	**No**	**Yes**	***P*-value†**	**No**	**Yes**	***P*-value†**	**No**	**Yes**	***P*-value†**	**No**	**Yes**	***P*-value†**
	**n (%)**	**n(%)**		**n (%)**	**n(%)**		**n (%)**	**n(%)**		**n (%)**	**n(%)**	
**Maternal age**			<0.001			0.786			<0.05			0.732
<20	987 (95.4)	48 (4.6)		846 (81.7)	189 (18.3)		819 (96.8)	27 (3.2)		817 (96.6)	29 (3.4)	
20-24	1759 (95.8)	78 (4.2)		1496 (81.4)	341 (18.6)		1460 (97.6)	36 (2.4)		1439 (96.2)	57 (3.8)	
25-29	1286 (93.3)	92 (6.7)		1132 (82.1)	246 (17.9)		1116 (98.6)	16 (1.4)		1094 (96.6)	38 (3.4)	
≥30	1159 (92.5)	94 (7.5)		1038 (82.8)	215 (17.2)		1022 (98.5)	16 (1.5)		1007 (97.0)	31 (3.0)	
**Parity**			0.121			<0.001			<0.001			0.222
0	1826 (94.9)	98 (5.1)		1468 (76.3)	456 (23.7)		1413 (96.3)	55 (3.7)		1408 (95.9)	60 (4.1)	
1-2	2878 (94.3)	175 (5.7)		2573 (84.3)	480 (15.7)		2535 (98.5)	38 (1.5)		2491 (96.8)	82 (3.2)	
≥3	487 (92.6)	39 (7.4)		471 (89.5)	55 (10.5)		469 (99.6)	2 (0.4)		458 (97.2)	13 (2.8)	
**Education (year)**			<0.01			<0.001			<0.01			0.739
0	659 (91.9)	58 (8.1)		639 (89.1)	78 (10.9)		633 (99.1)	6 (0.9)		620 (97.0)	19 (3.0)	
1-5	1440 (94.2)	88 (5.8)		1336 (87.4)	192 (12.6)		1317 (98.6)	19 (1.4)		1291 (96.6)	45 (3.4)	
>10	3092 (94.9)	166 (5.1)		2537 (77.9)	721 (22.1)		2467 (97.2)	70 (2.8)		2446 (96.4)	91 (3.6)	
**Wealth quintile**			0.293			<0.001			0.052			0.453
1- Poorest	771 (93.8)	51 (6.2)		727 (88.4)	95 (11.6)		715 (98.3)	12 (1.7)		705 (97.0)	22 (3.0)	
2	910 (94.7)	51 (5.3)		840 (87.4)	121 (12.6)		830 (98.8)	10 (1.2)		815 (97.0)	25 (3.0)	
3	1024 (94.9)	55 (5.1)		886 (82.1)	193 (17.9)		870 (98.2)	16 (1.8)		850 (95.9)	36 (4.1)	
4	1181 (95.0)	62 (5.0)		1019 (82.0)	224 (18.0)		993 (97.4)	26 (2.6)		978 (96.0)	41 (4.0)	
5-Wealthiest	1305 (93.3)	93 (6.7)		1040 (74.4)	358 (25.6)		1009 (97.0)	31 (3.0)		1009 (97.0)	31 (3.0)	

PIH, pregnancy-induced hypertension; CS: cesarean section; PPH, postpartum hemorrhage
†significance level by χ^2 tests
*analysis limited to all vaginal deliveri

Table S3. Association between background factors and pregnancy complications of PreSSMat cohort in Matlab, Bangladesh

	**PIH**			**CS**			**Perineal tear (2nd degree or more)***			**PPH***		
	**No**	**Yes**	***P*-value†**	**No**	**Yes**	***P*-value†**	**No**	**Yes**	***P*-value†**	**No**	**Yes**	***P*-value†**
	**n (%)**	**n(%)**		**n (%)**	**n(%)**		**n (%)**	**n(%)**		**n (%)**	**n(%)**	
**Maternal age**			<0.01			<0.01			<0.01			0.739
<20	652 (93.8)	43 (6.2)		373 (53.7)	322 (46.3)		365 (97.9)	8 (2.1)		367 (98.4)	6 (1.6)	
20-24	1111 (95.8)	49 (4.2)		558 (48.1)	602 (51.9)		536 (96.1)	22 (3.9)		546 (97.8)	12 (2.2)	
25-29	945 (96.1)	38 (3.9)		524 (53.3)	459 (46.7)		514 (98.1)	10 (1.9)		511 (97.5)	13 (2.5)	
≥30	868 (92.6)	69 (7.4)		524 (55.9)	413 (44.1)		520 (99.2)	4 (0.8)		510 (97.3)	14 (2.7)	
**Parity**			0.073			<0.001			0.560			0.182
0	1303 (93.9)	84 (6.1)		655 (47.2)	732 (52.8)		638 (97.4)	17 (2.6)		641 (97.9)	14 (2.1)	
1-2	2046 (95.4)	98 (4.6)		1163 (54.2)	981 (45.8)		1138 (97.9)	25 (2.1)		1139 (97.9)	24 (2.1)	
≥3	227 (93.0)	17 (7.0)		161 (66.0)	83 (34.0)		159 (98.8)	2 (1.2)		154 (95.7)	7 (4.3)	
**Education (year)**			0.926			<0.001			0.611			0.993
0	387 (95.1)	20 (4.9)		227 (55.8)	180 (44.2)		223 (98.2)	4 (1.8)		222 (97.8)	5 (2.2)	
1-5	624 (94.8)	34 (5.2)		404 (61.4)	254 (38.6)		397 (98.3)	7 (1.7)		395 (97.8)	9 (2.2)	
>10	2565 (94.6)	145 (5.4)		1348 (49.7)	1362 (50.3)		1315 (97.6)	33 (2.4)		1317 (97.7)	31 (2.3)	
**Wealth quintile**			0.097			<0.001			0.415			0.815
1- Poorest	563 (92.8)	44 (7.2)		388 (63.9)	219 (36.1)		379 (97.7)	9 (2.3)		377 (97.2)	11 (2.8)	
2	645 (96.0)	27 (4.0)		409 (60.9)	263 (39.1)		399 (97.6)	10 (2.4)		399 (97.6)	10 (2.4)	
3	712 (94.9)	38 (5.1)		391 (52.1)	359 (47.9)		382 (97.7)	9 (2.3)		384 (98.2)	7 (1.8)	
4	783 (95.4)	38 (4.6)		397 (48.4)	424 (51.6)		385 (97.0)	12 (3.0)		387 (97.5)	10 (2.5)	
5-Wealthiest	873 (94.4)	52 (5.6)		394 (42.6)	531 (57.4)		390 (99.0)	4 (1.0)		387 (98.2)	7 (1.8)	

PIH, pregnancy-induced hypertension; CS: cesarean section; PPH, postpartum hemorrhage
*analysis limited to all vaginal deliveries
†significance level by χ^2 tests

Table S4. Matrix indicating the association of background characteristics with outcomes in each cohort in Matlab, Bangladesh.

	MNCH cohort				PreSSMat cohort			
	Pregnancy-induced hypertension	Cesarean section	Perineal tear	Postpartum hemorrhage	Pregnancy-induced hypertension	Cesarean section	Perineal tear	Postpartum hemorrhage
Maternal age	√	x	√	x	√	√	√	x
Parity	√	√	√	x	√	√	x	x
Education	√	√	√	x	x	√	x	x
Wealth quintile	x	√	√	x	√	√	x	x

Table S5. Distribution of outcome of interest between included and not included in the analyses in two cohorts.

	**Pregnancy-induced hypertension**			**Cesarean section**			**Perineal tear**			**Postpartum hemorrhage**		
	Yes	No	*P*-value*	Yes	No	*P*-value*	Yes	No	*P*-value*	Yes	No	*P*-value*
	n (%)	n (%)		n (%)	n (%)		n (%)	n (%)		n (%)	n (%)	
Included in analysis	511 (5.5)	8767 (94.5)	0.749	2787 (30)	6491 (70)	<0.001	139 (2.1)	6352 (97.9)	0.594	200 (3.1)	6291 (96.9)	0.652
Not included in analysis	168 (5.4)	2968 (94.6)		686 (21.9)	2450 (78.1)		57 (2.3)	2393 (97.7)		71 (2.9)	2379 (97.1)	

*level of significance by χ^2 test
